# Supplementary material for: Cadherin preserves cohesion across involuting tissues during C. elegans neurulation
Source: eLife. 2020 Oct 8;9:e58626. doi: 10.7554/eLife.58626 (PMC7544503; doi:10.7554/eLife.58626)
Supplement: Supplementary file 1. [file elife-58626-supp1.docx]

**Supplementary File 1: List of Neurons Used in Figure Panels**

|  | Fig 1a, b | Fig 4c |  | Fig 1a, b | Fig 4c |  | Fig 1a, b | Fig 4c |
| --- | --- | --- | --- | --- | --- | --- | --- | --- |
| AIAL | x |  | IL2DL | x |  | RMGR | x |  |
| AIAR | x |  | IL2DR | x |  | SAADL | x |  |
| AIML | x | x | IL2L | x |  | SAADR | x |  |
| AIMR | x | x | IL2R | x |  | SAAVL | x | x |
| AINL | x | x | IL2VL | x |  | SAAVR | x | x |
| AINR | x | x | IL2VR | x |  | SABDL | x |  |
| AIYL | x | x | OLLL | x |  | SABDR | x |  |
| AIYR | x | x | OLLR | x |  | SABVL | x |  |
| ALA | x | x | OLQDL | x |  | SABVR | x |  |
| AVAL | x | x | OLQDR | x |  | SIADL | x | x |
| AVAR | x | x | OLQVL | x |  | SIADR | x | x |
| AVDL | x | x | OLQVR | x |  | SIAVL | x |  |
| AVDR | x | x | RIAL | x |  | SIAVR | x |  |
| AVEL | x |  | RIAR | x |  | SIBVL | x | x |
| AVER | x |  | RID | x |  | SIBVR | x | x |
| AVG | x |  | RIFL | x |  | SMBDL | x |  |
| AVHL | x | x | RIFR | x |  | SMBDR | x |  |
| AVHR | x | x | RIGL | x |  | SMBVL | x |  |
| AVJL | x |  | RIGR | x |  | SMBVR | x |  |
| AVJR | x |  | RIH | x |  | SMDDL | x | x |
| AVKL | x |  | RIPL | x | x | SMDDR | x | x |
| AVKR | x |  | RIPR | x | x | SMDVL | x | x |
| AVL | x | x | RIR | x |  | SMDVR | x | x |
| BAGL | x |  | RIS | x |  | URAVL | x | x |
| BAGR | x |  | RIVL | x | x | URAVR | x | x |
| CEMVL | x | x | RIVR | x | x | URADL | x |  |
| CEMVR | x | x | RMDDL | x |  | URADR | x |  |
| CEPDL | x |  | RMDDR | x |  | URBL | x |  |
| CEPDR | x |  | RMDL | x |  | URBR | x |  |
| CEPVL | x |  | RMDR | x | x | URXL | x |  |
| CEPVR | x |  | RMDVL | x | x | URXR | x |  |
| IL1DL | x |  | RMDVR | x |  | URYDL | x |  |
| IL1DR | x |  | RMED | x | x | URYDR | x |  |
| IL1L | x |  | RMEL | x | x | URYVL | x | x |
| IL1R | x |  | RMER | x | x | URYVR | x | x |
| IL1VL | x |  | RMEV | x |  |  |  |  |
| IL1VR | x |  | RMGL | x |  |  |  |  |
